# Unilateral Arm Crank Exercise Test for Assessing Cardiorespiratory Fitness in Individuals with Hemiparetic Stroke

**DOI:** 10.1155/2017/6862041

**Published:** 2017-12-31

**Authors:** Kazuaki Oyake, Tomofumi Yamaguchi, Chihiro Oda, Daisuke Kudo, Kunitsugu Kondo, Yohei Otaka, Kimito Momose

**Affiliations:** ^1^Department of Physical Therapy, School of Health Sciences at Narita, International University of Health and Welfare, 4-3 Kozunomori, Narita-shi, Chiba 286-8686, Japan; ^2^Japan Society for the Promotion of Science, 5-3-1 Kojimachi, Chiyoda-ku, Tokyo 102-0083, Japan; ^3^Department of Physical Therapy, Yamagata Prefectural University of Health Sciences, 260 Kamiyanagi, Yamagata-shi, Yamagata 990-2212, Japan; ^4^Department of Neuroscience, University of Copenhagen, Nørregade 10, 1165 København, Denmark; ^5^Department of Rehabilitation Medicine, Keio University School of Medicine, 35 Shinanomachi, Shinjuku-ku, Tokyo 160-0016, Japan; ^6^Graduate School of Sport Sciences, Waseda University, 2-579-15 Mikajima, Tokorozawa-shi, Saitama 359-1192, Japan; ^7^Tokyo Bay Rehabilitation Hospital, 4-1-1 Yatsu, Narashino-shi, Chiba 275-0026, Japan; ^8^Department of Rehabilitation Medicine I, School of Medicine, Fujita Health University, 1-98 Dengakugakubo, Kutsukake-cho, Toyoake-shi, Aichi 470-1192, Japan; ^9^Department of Physical Therapy, School of Health Sciences, Shinshu University, 3-1-1 Asahi, Matsumoto-shi, Nagano 390-8621, Japan

## Abstract

Cardiorespiratory fitness assessment with leg cycle exercise testing may be influenced by motor impairments in the paretic lower extremity. Hence, this study examined the usefulness of a unilateral arm crank exercise test to assess cardiorespiratory fitness in individuals with stroke, including sixteen individuals with hemiparetic stroke (mean ± SD age, 56.4 ± 7.5 years) and 12 age- and sex-matched healthy controls. Participants performed the unilateral arm crank and leg cycle exercise tests to measure oxygen consumption ($\dot{V}$O_2_) and heart rate at peak exercise. The $\dot{V}$O_2_ at peak exercise during the unilateral arm crank exercise test was significantly lower in the stroke group than in the control group (*p* < 0.001). In the stroke group, the heart rate at peak exercise during the unilateral arm crank exercise test did not significantly correlate with the Brunnstrom recovery stages of the lower extremity (*p* = 0.137), whereas there was a significant correlation during the leg cycle exercise test (rho = 0.775, *p* < 0.001). The unilateral arm crank exercise test can detect the deterioration of cardiorespiratory fitness independently of lower extremity motor impairment severity in individuals with hemiparetic stroke. This study is registered with UMIN000014733.

## 1. Introduction

Cardiorespiratory fitness in individuals with stroke is reduced to 26–87% of that in age- and sex-matched healthy persons [1]. Even independently ambulant and community dwelling individuals with stroke have reduced cardiorespiratory fitness as compared to nonstroke individuals [2]. Cardiorespiratory fitness reduction is potentially related to walking disability [3, 4], limitations in activities of daily living [5–7], and an increased risk of further cardiovascular disease [8] in individuals with stroke. In addition, cardiorespiratory fitness is associated with better cognitive performance, greater grey matter volume, and greater integrity of the white matter in individuals with stroke [9]. Therefore, the assessment of cardiorespiratory fitness is essential for identifying physical deconditioning, predicting prognosis, and assessing the effects of therapeutic exercise in stroke rehabilitation [10, 11].

Oxygen consumption ($\dot{V}$O_2_) at peak exercise measured during the leg cycle exercise test is commonly used to assess cardiorespiratory fitness in individuals with hemiparetic stroke [12–16]. However, with this approach, motor impairments in the paretic lower extremity may limit exercise test performance [13–15]. Considering the possibility that the reduced $\dot{V}$O_2_ at peak exercise in individuals with hemiparetic stroke reflects motor impairments in the paretic lower extremity, development of a cardiorespiratory fitness assessment that is not influenced by motor impairments is required [17].

An arm crank ergometer can be used for assessing cardiorespiratory fitness, particularly in individuals with motor impairments in the paretic lower extremity, such as in spinal cord injury [18]. However, the conventional bilateral arm crank ergometer is not suitable for individuals with hemiparetic stroke, because of the limitations associated with hemiparesis. Here, a unilateral arm crank exercise test was employed as a unique strategy [19, 20]. Unilateral arm crank exercise testing with the nonparetic arm in individuals with stroke is potentially useful to assess cardiorespiratory fitness independently of lower extremity motor impairment severity. However, there are no reports that have compared cardiorespiratory fitness in individuals with stroke and healthy adults using the unilateral arm crank exercise test. In addition, no study has directly examined the relationship between heart rate at peak exercise during the unilateral arm crank exercise test and motor impairments of the paretic lower extremity. This study aimed to examine the usefulness of a unique exercise test performed with the nonparetic arm for assessing cardiorespiratory fitness in individuals with hemiparetic stroke. We hypothesized that the unilateral arm crank exercise test can detect the deterioration of cardiorespiratory fitness in individuals with hemiparetic stroke. We also hypothesized that unilateral arm crank exercise testing with the nonparetic arm can assess cardiorespiratory fitness independently of the lower extremity motor impairment severity in individuals with hemiparetic stroke.

## 2. Methods

### 2.1. Study Design

This study used a cross-sectional observational design. The study protocol was approved by the appropriate ethics committee. All participants provided written informed consent prior to study enrollment. The study was conducted according to the Declaration of Helsinki of 1964, as revised in 2013.

### 2.2. Participants

Sixteen individuals with hemiparetic stroke participated in this study. A group of 12 healthy volunteers matched for age and sex participated as controls. Participants with stroke were recruited from a convalescent rehabilitation hospital between November 2014 and November 2015. Control participants were recruited from a local community. The inclusion criteria in participants with stroke were as follows: age 40–80 years, being within 180 days after first-ever stroke, ability to remain seated independently for 30 min without any support, and a Mini-Mental State Examination score [21] of 24 or more. The inclusion criteria in control participants were as follows: age 40–80 years, a Mini-Mental State Examination score of 24 or more, and no involvement in regular exercise more than twice a week. The exclusion criteria in both participants with stroke and control participants were as follows: limited range of motion and/or pain that could affect the exercise test, unstable medical conditions, such as unstable angina, uncontrolled hypertension, or tachycardia, use of beta-blockers, and any comorbid neurological disorder.

### 2.3. Exercise Testing

All participants performed the unilateral arm crank and leg cycle exercise tests on different days, but within 7 days. The order of the two tests was randomly determined for each participant to remove order bias. Both tests were performed on an ergometer (Strength Ergo 240, Mitsubishi Electric Engineering Co., Tokyo) that can be precisely load-controlled (coefficient of variation, 5%) over a wide range of pedaling resistance (0–400 W). Participants were instructed not to eat food for 3 h and to avoid caffeine and vigorous physical activity for at least 6 and 24 h, respectively, before the tests [22].

**Figure 1:**
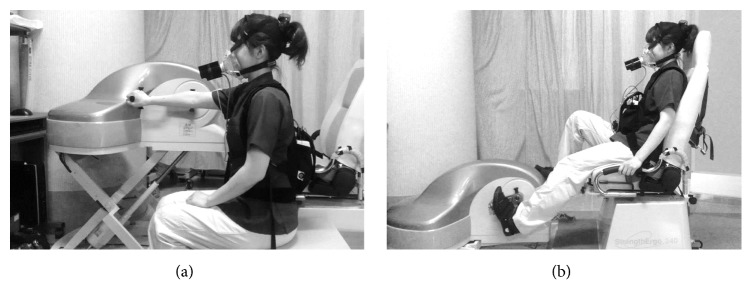
Experimental setup of the unilateral arm crank exercise test (a) and the leg cycle exercise test (b).

For the unilateral arm crank exercise test, the rotational axis of the ergometer was set at the height of participant's shoulder [23] and at a distance where the participant's elbow was in a slightly bent position with maximal reach (Figure 1(a)). The resistance was set at 10 W for the first 3 min of exercise testing and gradually increased by 5 W·min^−1^ in both the stroke and control groups [23].

For the leg cycle exercise test, the distance from the seat edge to pedal axis was adjusted so that the participant's knee flexion angle was 20° when extended maximally (Figure 1(b)). The backrest was set at 20° reclined from the vertical position. For participants with stroke, additional strapping was attached to secure the paretic foot to the pedal as needed. The resistance was set at 10 W for the first 3 min of exercise testing and was gradually increased by 10 W·min^−1^ in both groups [15].

Control participants were instructed to maintain a target cadence of 50 rpm throughout the exercise in both tests [15, 23]. Each participant with stroke was instructed to maintain a speed at which they could perform the test comfortably at 10 W (37 ± 7 rpm in the unilateral arm crank exercise test and 46 ± 7 rpm in the leg cycle exercise test) throughout each test, because some participants could not maintain a cadence of 50 rpm, even at 10 W.

Criteria for termination of either exercise test included one of the following: the participant reached 85% of the age-predicted maximal heart rate (220 minus age) [24], the participant was unable to maintain a target cadence, or the participant appeared to be in distress as defined in the termination criteria established by the American College of Sports Medicine [22].

An expired gas analyzer (Aerosonic AT-1100, ANIMA Corp., Tokyo) and a heart rate monitor (Polar WearLink, Polar Electro Japan Inc., Tokyo) were used to measure the expired gas and heart rate, respectively, during the exercise tests. Expired gas and heart rate were measured simultaneously on a breath-by-breath basis. The expired gas data were smoothed with a 30 s moving average to minimize breath-to-breath variability in determining the $\dot{V}$O_2_ values at peak exercise [14, 25]. The $\dot{V}$O_2_, heart rate, respiratory exchange ratio, minute ventilation, the ventilatory equivalents of oxygen and carbon dioxide, and the end-tidal oxygen and carbon dioxide at peak exercise were defined as the highest values achieved during exercise testing [14]. The heart rate at peak exercise is an important determining factor for the $\dot{V}$O_2_ at peak exercise and is an indicator of the degree of effort [24]. The respiratory exchange ratio is defined as the ratio between carbon dioxide output ($\dot{V}$CO_2_) and $\dot{V}$O_2_ and is also an indicator of exercise effort [24]. Work rate at peak exercise was defined as the highest work rate maintained for at least 30 s [24]. Participants provided their ratings of perceived exertion (6 = no exertion at all, 20 = maximal exertion) [26] at the end of the test. Participants who discontinued exercise testing because of their inability to maintain a target cadence were requested to report the reason (i.e., either general fatigue or limb muscle fatigue). A 3-lead electrocardiogram (BSM-2401, Nihon Kohden Corp., Tokyo) was used to monitor cardiac activity throughout the tests and during the recovery phase. Blood pressure was obtained every minute from the paretic arm in the stroke group and the nondominant arm in the control group using an automated system (Tango, Sun Tech Medical Inc., NC).

The ventilatory threshold was determined using a combination of the following criteria: (1) the point where the ventilatory equivalent of oxygen reaches its minimum or starts to increase, without an increase in the ventilatory equivalent of carbon dioxide; (2) the point at which the end-tidal oxygen fraction reaches a minimum or starts to increase, without a decline in the end-tidal carbon dioxide fraction; (3) the point of deflection of $\dot{V}$CO_2_ versus $\dot{V}$O_2_ (V-slope method) [27]. The first two criteria were prioritized in case the three criteria presented different results [28]. The ventilatory threshold was determined as the averages from two independent raters (CO and DK), when the difference in the $\dot{V}$O_2_ values of the corresponding points as determined by the two raters was less than 100 mL·min^−1^ [29]. In case of any discrepancy, a third experienced rater (KO) judged the point, and the ventilatory threshold was taken as the average of the two closest values [28]. The $\dot{V}$O_2_ at the ventilatory threshold was used as a submaximal index of exercise capacity [24].

### 2.4. Motor Impairment Assessment

The Brunnstrom recovery stages [30] consisted of six categories: stage I, flaccid; stage II, synergy pattern development with minimal voluntary movement; stage III, voluntary synergistic movement; stage IV, some movements deviating from synergy; stage V, independent movement apart from the basic synergic pattern; and stage VI, isolated voluntary joint movements.

### 2.5. Statistical Analysis

Participant characteristics between the stroke and control groups were compared using unpaired *t*-test for continuous variables and Fisher's exact test for dichotomous variables, respectively. The $\dot{V}$O_2_ differences between the stroke and control groups in each test were examined using the unpaired *t*-test to determine if the tests could detect the $\dot{V}$O_2_ reduction in individuals with stroke. The Spearman rank correlation coefficient was used to examine whether the heart rate and $\dot{V}$O_2_ at peak exercise correlated with motor impairments in the paretic lower extremity. Furthermore, heart rate, respiratory exchange ratio, minute ventilation, the ventilatory equivalents of oxygen and carbon dioxide, the end-tidal oxygen and carbon dioxide, work rate, systolic and diastolic blood pressures, ratings of perceived exertion, and $\dot{V}$O_2_ at the ventilatory threshold were compared between stroke and control groups using the unpaired *t*-test or the Mann–Whitney *U* test, depending on the type of variable. To identify whether maximal effort was achieved during the exercise test, we set the following criteria [24]: (1) the exercise test was terminated when achieving 85% of the age-predicted maximal heart rate and (2) the respiratory exchange ratio at peak exercise was 1.10 or more. The number of participants who met a criterion for maximal effort was compared between groups using Fisher's exact test. Statistical analyses were performed using the Statistical Package for the Social Sciences software version 21.0 (International Business Machines Corp., NY). Any *p* values < 0.05 were considered statistically significant.

## 3. Results

Of the 90 individuals who met the inclusion criteria, 74 were excluded based on the exclusion criteria; consequently, 16 individuals with stroke participated in the study.  Table 1 shows the characteristics of the 16 participants. There were no significant differences in age, sex, height, weight, and body mass index between the stroke and control groups (*p* > 0.05).

No significant adverse events occurred during or after either exercise test in both the stroke and control groups. Measurement values at peak exercise in each group during the unilateral arm crank and leg cycle exercise tests are shown in Tables 2 and 3, respectively. The mean $\dot{V}$O_2_ at peak exercise during the unilateral arm crank exercise test in the stroke group was significantly reduced to 73.0% (mean difference = −3.7; 95% confidence interval [CI] = −5.6, −1.7; *p* < 0.001) of that in the control group. In the leg cycle exercise test, the mean $\dot{V}$O_2_ at peak exercise in the stroke group was also reduced to 66.3% (mean difference = −6.5; 95% CI = −9.8, −3.2; *p* < 0.001) of that in the control group. Results showed that the unilateral arm crank exercise test, as well as the leg cycle exercise test, detected the deterioration of cardiorespiratory fitness in participants with hemiparetic stroke compared with healthy controls.

**Figure 2:**
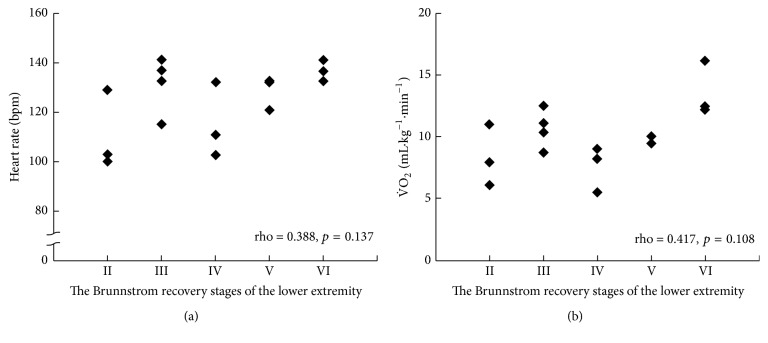
Correlations between heart rate and the Brunnstrom recovery stages of the lower extremity (a) and that between $\dot{V}$O_2_ and the Brunnstrom recovery stages of the lower extremity (b) as measured during the unilateral arm crank exercise test.

**Figure 3:**
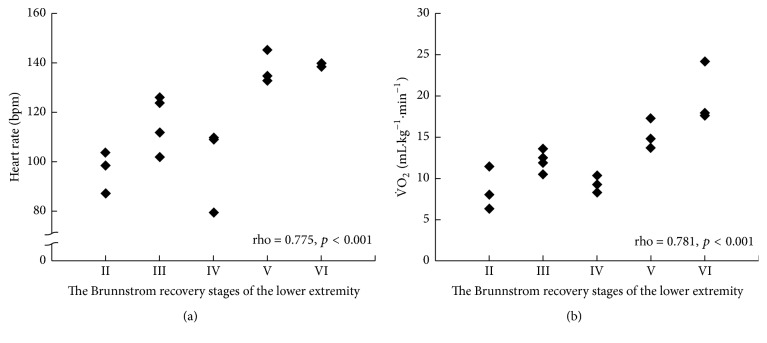
Correlations between heart rate and the Brunnstrom recovery stages of the lower extremity (a) and that between $\dot{V}$O_2_ and the Brunnstrom recovery stages of the lower extremity (b) as measured during the leg cycle exercise test.

The heart rate and $\dot{V}$O_2_ at peak exercise during the unilateral arm crank exercise test did not correlate with the Brunnstrom recovery stages of the lower extremity (rho = 0.388, 95% CI = −0.133, 0.741, and *p* = 0.137; rho = 0.417, 95% CI = −0.099, 0.756, and *p* = 0.108, resp.) (Figure 2), whereas those during the leg cycle exercise test correlated significantly (rho = 0.775, 95% CI = 0.454, 0.918, and *p* < 0.001; rho = 0.781, 95% CI = 0.466, 0.920, and *p* < 0.001, resp.) (Figure 3).

The heart rate and respiratory exchange ratio at peak exercise during the unilateral arm crank exercise test were not significantly different between the stroke and control groups (mean difference = −4, 95% CI = −14, 6, and *p* = 0.428; mean difference = 0.13, 95% CI = −0.03, 0.30, and *p* = 0.102, resp.). Six participants with hemiparetic stroke (37.5%) and 5 control participants (41.7%) achieved 85% of the age-predicted maximal heart rate during the exercise test (*p* = 0.999). The respiratory exchange ratio at peak exercise of 1.10 or more was observed in 14 participants with hemiparetic stroke (87.5%) and in 7 control participants (58.3%) (*p* = 0.103). In addition, the work rate at peak exercise during the unilateral arm crank exercise was also not significantly different between the groups (mean difference = −2.8; 95% CI = −17.0, 11.3; *p* = 0.702). These results showed that both the stroke and control groups could achieve the same exercise intensity level during the unilateral arm crank exercise test. The ventilatory equivalents of oxygen and carbon dioxide at peak exercise during the unilateral arm crank exercise test were significantly higher in the stroke group than in the control group (mean difference = 12.9, 95% CI = 3.9, 21.9, and *p* = 0.007; mean difference = 6.6, 95% CI = 0.9, 12.2, and *p* = 0.024, resp.). The end-tidal oxygen fraction at peak exercise during the unilateral arm crank exercise test was also significantly higher in the stroke group than in the control group (mean difference = 1.0; 95% CI = 0.3, 1.6; *p* = 0.006). The ventilatory threshold was identifiable in all participants during the unilateral arm crank exercise test. The $\dot{V}$O_2_ at the ventilatory threshold was significantly lower in the stroke group than in the control group (mean difference = −2.8; 95% CI = −3.8, −1.7; *p* < 0.001).

The heart rate at peak exercise during the leg cycle exercise test was significantly lower in the stroke group than in the control group (mean difference = −21; 95% CI = −32, −8; *p* = 0.003), whereas there was no significant difference in the respiratory exchange ratio at peak exercise between the groups (mean difference = 0.05; 95% CI = −0.06, 0.18; *p* = 0.336). Six participants with hemiparetic stroke (37.5%) and all the control participants (100.0%) achieved 85% of the age-predicted maximal heart rate during the exercise test (*p* < 0.001). Only 3 participants with hemiparetic stroke reached 85% of the age-predicted maximal heart rate in both the unilateral arm crank and leg cycle exercise tests. The respiratory exchange ratio at peak exercise of 1.10 or more was observed in 9 participants with hemiparetic stroke (56.3%) and in 8 control participants (66.7%) (*p* = 0.705). Nine participants with hemiparetic stroke reached the respiratory exchange ratio at peak exercise of 1.10 or more in both exercise tests. The work rate at peak exercise during the leg cycle exercise was significantly lower in the stroke group than in the control group (mean difference = −37.4; 95% CI = −60.4, −14.5; *p* = 0.004). The ventilatory equivalents of oxygen and carbon dioxide at peak exercise during the leg cycle exercise test were significantly higher in the stroke group than in the control group (mean difference = 6.8, 95% CI = 1.8, 11.8, and *p* = 0.009; mean difference = 6.1, 95% CI = 1.4, 11.0, and *p* = 0.014, resp.). The end-tidal oxygen fraction at peak exercise during the leg cycle exercise test was also significantly higher in the stroke group than in the control group (mean difference = 0.9; 95% CI = 0.1, 1.7; *p* = 0.028). The ventilatory threshold was identifiable in all participants during the leg cycle exercise test. The $\dot{V}$O_2_ at the ventilatory threshold was significantly lower in the stroke group compared with the control group (mean difference = −5.2; 95% CI = −7.1, −3.4; *p* < 0.001).

## 4. Discussion

This is the first study to evaluate the usefulness of unilateral arm crank exercise test for assessing cardiorespiratory fitness in individuals with hemiparetic stroke. All participants completed the unilateral arm crank exercise test using their nonparetic arm without any adverse events. The $\dot{V}$O_2_ at peak exercise and the $\dot{V}$O_2_ at the ventilatory threshold during the unilateral arm crank exercise test were significantly reduced in the stroke group compared with the control group. Moreover, the heart rate at peak exercise during the unilateral arm crank exercise test did not correlate with the Brunnstrom recovery stages of the lower extremity. These results suggest that the unilateral arm crank exercise test can detect the deterioration of cardiorespiratory fitness independently of the lower extremity motor impairment severity in individuals with hemiparetic stroke.

Tang et al. [15] reported that motor impairments in the paretic lower extremity may limit exercise test performance during the leg cycle exercise test. In this study, all control participants and only 37.5% (*n* = 6) of the participants with hemiparetic stroke achieved 85% of the age-predicted maximal heart rate during the leg cycle exercise test. In addition to heart rate, there was a significant correlation between the $\dot{V}$O_2_ at peak exercise and the Brunnstrom recovery stages of the lower extremity during the leg cycle exercise test. Furthermore, both the heart rate and $\dot{V}$O_2_ at peak exercise during the leg cycle exercise test were significantly lower in the stroke group than in the control group. These results indicate that the Brunnstrom recovery stages of lower extremity scores may be a covariate in exploring the differences between individuals with stroke and healthy adults using the leg cycle exercise test. Conversely, there was no significant correlation between the $\dot{V}$O_2_ at peak exercise and the Brunnstrom recovery stages of the lower extremity during the unilateral arm crank exercise test. These findings suggest that assessment of cardiorespiratory fitness using the unilateral arm crank exercise test can be applied to individuals with stroke independently of motor impairment severity in the paretic lower extremity. Additionally, compared with the control group, the stroke group exhibited a reduction in the $\dot{V}$O_2_ at peak exercise during the unilateral arm crank exercise test but no difference in the heart rate. Considering that $\dot{V}$O_2_ is the product of heart rate, stroke volume, and arterial-venous oxygen difference [24], the reduced $\dot{V}$O_2_ at peak exercise in participants with stroke observed during the unilateral arm crank exercise test may represent the decline in the stroke volume and/or arterial-venous oxygen difference at peak exercise.

A few concerns with the unilateral arm crank exercise test were identified in this study. In both the stroke and control groups, over 50.0% (*n* = 10 in the stroke group and *n* = 7 in the control group) of the participants had difficulty achieving 85% of the age-predicted maximal heart rate during the unilateral arm crank exercise. Five out of 16 participants with hemiparetic stroke (31.3%) and 4 out of 12 control participants (33.3%) discontinued the unilateral arm crank exercise because of arm muscle fatigue, which could be attributed to the greater recruitment of metabolically inefficient type II muscle fibers during unilateral arm cranking [31]. Moreover, a few participants had a diastolic blood pressure greater than 115 mmHg during the unilateral arm exercise test. di Blasio et al. [32] suggested that small muscle mass exercises, such as arm cranking, generate increased intramuscular pressure, which reduces muscular perfusion and increases resistance to blood circulation. Thus, the protocol in the present study might result in an overload on their arm muscle rather than the cardiorespiratory function. Despite concerns regarding local fatigue, as stated above, a respiratory exchange ratio at peak exercise of 1.10 or more was observed in 14 participants with hemiparetic stroke (87.5%) and in 7 control participants (58.3%) during the unilateral arm crank exercise test in the present study. It has previously been reported that a respiratory exchange ratio of 1.10 or more is generally an indication of excellent participant effort during exercise tests [24]. Therefore, the results may support consideration that the unilateral arm crank exercise test can detect the $\dot{V}$O_2_ response at maximal effort. Although further studies are required to determine more appropriate protocols (e.g., target cadence, increments in exercise intensity, or target heart rate) for the unilateral arm crank exercise test, it should be noted that the present feasibility study is the first to show the usefulness of a unilateral arm crank exercise test for assessing cardiorespiratory fitness in individuals with hemiparetic stroke, as it could detect a decline in cardiorespiratory fitness independently of lower extremity motor impairment severity in individuals with stroke.

In both the unilateral arm crank and leg cycle exercise tests, there was no significant difference in the minute ventilation at peak exercise between the stroke and control groups, although the $\dot{V}$O_2_ at peak exercise was significantly lower in the stroke group than in the control group. In addition, the ventilation equivalents of oxygen and carbon dioxide, and the end-tidal oxygen fraction at peak exercise, were significantly higher in the stroke group than in the control group. Therefore, ventilatory efficiency may be compromised in individuals with hemiparetic stroke during the unilateral arm crank and leg cycle exercise tests. Moreover, Sisante et al. [33] have reported that individuals with subacute stroke have low ventilatory efficiency when cardiorespiratory exercise testing was performed using a recumbent stepper. The unilateral arm crank exercise test also provides useful information on ventilatory efficiency as well as on deterioration in cardiorespiratory fitness in individuals with hemiparetic stroke.

This study had several limitations. First, it included a relatively small sample of individuals with subacute stroke; therefore, generalization of the findings should be made with caution. Second, normative values for the cardiorespiratory fitness assessment using the unilateral arm crank exercise test remain unclear. Establishing standard values and minimal detectable change in variables measured during the unilateral arm crank exercise test in healthy adults and individuals with stroke is necessary. Finally, previous studies showed that a total-body recumbent stepper exercise test [33–35], a nonparetic leg cycle exercise test [36, 37], and a robotics-assisted tilt table exercise test [28, 38] may be useful for assessing cardiorespiratory fitness in individuals with hemiparetic stroke. In future studies, evaluating the advantages of a unilateral arm crank exercise test over these exercise tests for assessing cardiorespiratory fitness in individuals with hemiparetic stroke is warranted.

## 5. Conclusions

This study suggests that the unilateral arm crank exercise test can detect cardiorespiratory fitness deterioration in individuals with hemiparetic stroke. The test could also assess cardiorespiratory fitness independently of lower extremity motor impairment severity in individuals with hemiparetic stroke. Therefore, the unilateral arm crank exercise test may be useful for assessing cardiorespiratory fitness in individuals with hemiparetic stroke.

## Figures and Tables

**Table 1:** Participant characteristics.

Variable	Stroke (*n* = 16)	Control (*n* = 12)	95% CI	*p* value
Age (years), mean ± SD	56.4 ± 7.3	58.3 ± 7.8	−7.7, 4.1	0.535
Sex, *n* (%)				
Men	11 (68.8)	4 (33.3)	NA	0.125
Women	5 (31.2)	8 (66.7)		
Height (m), mean ± SD	1.66 ± 0.09	1.61 ± 0.11	−0.03, 0.12	0.206
Weight (kg), mean ± SD	60.3 ± 10.7	59.7 ± 12.1	−8.2, 9.6	0.877
Body mass index (kg/m^2^), mean ± SD	21.9 ± 2.9	22.9 ± 2.9	−3.2, 1.3	0.406
Antihypertensive medications, *n* (%)				
Angiotensin-converting enzyme inhibitor	3 (18.8)	0 (0.0)	NA	<0.001
Angiotensin II receptor blocker	5 (31.2)	0 (0.0)		
Calcium channel blocker	11 (68.8)	0 (0.0)		
Comorbidities, *n* (%)				
Hypertension	14 (87.5)	0 (0.0)	NA	<0.001
Diabetes mellitus	5 (31.2)	0 (0.0)		
Hyperlipidemia	3 (18.8)	0 (0.0)		
Type of stroke, *n* (%)				
Ischemic	5 (31.2)	NA		
Hemorrhagic	11 (68.8)			
Side affected by stroke, *n* (%)				
Right	6 (37.5)	NA		
Left	10 (62.5)			
Time since stroke (days), mean ± SD	101 ± 39	NA		
Brunnstrom recovery stages of lower extremity, *n* (%)				
II	3 (18.7)	NA		
III	4 (25.0)			
IV	3 (18.7)			
V	3 (18.7)			
VI	3 (18.7)			

95% CI: 95% confidence interval of the difference between the means (stroke group − control group), NA: not applicable.

**Table 2:** Comparisons of various parameters at peak exercise in participants with stroke and healthy controls in the unilateral arm crank exercise test.

Variable	Unilateral arm crank exercise test			
	Stroke	Control	95% CI	*p* value
	(*n* = 16)	(*n* = 12)		
$\dot{V}$O_2_ (mL·kg^−1^·min^−1^), mean ± SD	10.0 ± 2.6	13.7 ± 2.1	−5.6, −1.7	<0.001
Heart rate (bpm), mean ± SD	125 ± 14	129 ± 11	−14, 6	0.428
Respiratory exchange ratio, mean ± SD	1.28 ± 0.25	1.15 ± 0.13	−0.03, 0.30	0.102
Minute ventilation (L·min^−1^), mean ± SD	28.2 ± 9.0	29.5 ± 9.1	−8.4, 5.8	0.715
Ventilatory equivalent of oxygen, mean ± SD	48.1 ± 14.7	35.2 ± 3.6	3.9, 21.9	0.007
Ventilatory equivalent of carbon dioxide, mean ± SD	39.9 ± 9.0	33.3 ± 3.5	0.9, 12.2	0.024
End-tidal oxygen fraction (%), mean ± SD	17.1 ± 1.0	16.1 ± 0.5	0.3, 1.6	0.006
End-tidal carbon dioxide fraction (%), mean ± SD	5.01 ± 0.66	5.32 ± 0.51	−0.76, 0.14	0.166
Work rate (*W*), mean ± SD	34.1 ± 22.7	36.9 ± 7.4	−17.0, 11.3	0.702
Systolic blood pressure (mmHg), mean ± SD	177 ± 17	192 ± 18	−29, −1	0.043
Diastolic blood pressure (mmHg), mean ± SD	102 ± 13	105 ± 13	−17, 11	0.484
Ratings of perceived exertion, median (IQR)	15 (14, 17)	15 (13, 17)	NA	0.504
$\dot{V}$O_2_ at the ventilatory threshold (mL·kg^−1^·min^−1^), mean ± SD	7.4 ± 1.2	10.2 ± 1.6	−3.8, −1.7	<0.001
Number of participants who terminated exercise test when achieving 85% of the age-predicted maximal heart rate, *n* (%)	6 (37.5)	5 (41.7)	NA	0.999
Number of participants who achieved the respiratory exchange ratio at peak exercise of 1.10 or more, *n* (%)	14 (87.5)	7 (58.3)	NA	0.103
Termination reasons prior to achieving 85% of the age-predicted maximal heart rate, *n* (%)				
General fatigue	3 (18.8)	0 (0.0)		
Arm muscle fatigue	5 (31.2)	4 (33.3)		
Diastolic blood pressure > 115 mmHg	2 (12.5)	3 (25.0)		

95% CI: 95% confidence interval of the difference between the means (stroke group − control group), IQR: interquartile range, NA: not applicable.

**Table 3:** Comparisons of various parameters at peak exercise in participants with stroke and healthy controls in the leg cycle exercise test.

Variable	Leg cycle exercise test			
	Stroke	Control	95% CI	*p* value
	(*n* = 16)	(*n* = 12)		
$\dot{V}$O_2_ (mL·kg^−1^·min^−1^), mean ± SD	13.0 ± 4.6	19.6 ± 3.6	−9.8, −3.2	<0.001
Heart rate (bpm), mean ± SD	117 ± 20	138 ± 7	−32, −8	0.003
Respiratory exchange ratio, mean ± SD	1.15 ± 0.18	1.10 ± 0.12	−0.06, 0.18	0.336
Minute ventilation (L·min^−1^), mean ± SD	29.6 ± 13.1	36.7 ± 11.8	−17.0, 2.7	0.150
Ventilatory equivalent of oxygen, mean ± SD	38.3 ± 7.2	31.5 ± 5.0	1.8, 11.8	0.009
Ventilatory equivalent of carbon dioxide, mean ± SD	36.0 ± 7.4	29.9 ± 3.7	1.4, 11.0	0.014
End-tidal oxygen fraction (%), mean ± SD	16.4 ± 1.1	15.5 ± 0.9	0.1, 1.7	0.028
End-tidal carbon dioxide fraction (%), mean ± SD	5.51 ± 0.75	5.99 ± 0.62	−1.0, 0.1	0.081
Work rate (*W*), mean ± SD	55.0 ± 34.6	92.4 ± 9.2	−60.4, −14.5	0.004
Systolic blood pressure (mmHg), mean ± SD	164 ± 24	187 ± 16	−38, −7	0.009
Diastolic blood pressure (mmHg), mean ± SD	88 ± 14	93 ± 16	−17, 6	0.363
Ratings of perceived exertion, median (IQR)	13 (13, 15)	13 (13, 15)	NA	0.923
$\dot{V}$O_2_ at the ventilatory threshold (mL·kg^−1^·min^−1^), mean ± SD	9.5 ± 2.2	14.7 ± 2.6	−7.1, −3.4	<0.001
Number of participants who terminated exercise test when achieving 85% of the age-predicted maximal heart rate, *n* (%)	6 (37.5)	12 (100.0)	NA	<0.001
Number of participants who achieved the respiratory exchange ratio at peak exercise of 1.10 or more, *n* (%)	9 (56.3)	8 (66.7)	NA	0.705
Termination reasons prior to achieving 85% of the age-predicted maximal heart rate, *n* (%)				
General fatigue	4 (25.0)	0 (0.0)		
Leg muscle fatigue	5 (31.2)	0 (0.0)		
Diastolic blood pressure > 115 mmHg	0 (0.0)	0 (0.0)		
Ankle clonus	1 (6.3)	0 (0.0)		

95% CI: 95% confidence interval of the difference between the means (stroke group − control group), IQR: interquartile range, NA: not applicable.
